# Hypoxia enhances IPF mesenchymal progenitor cell fibrogenicity via the lactate/GPR81/HIF1**α** pathway

**DOI:** 10.1172/jci.insight.163820

**Published:** 2023-02-22

**Authors:** Libang Yang, Adam Gilbertsen, Hong Xia, Alexey Benyumov, Karen Smith, Jeremy Herrera, Emil Racila, Peter B. Bitterman, Craig A. Henke

**Affiliations:** 1 Department of Medicine and; 2 CSENG Biomedical Engineering, University of Minnesota, Minneapolis, Minnesota, USA.; 3 Department of Laboratory Medicine and Pathology, Minneapolis, Minnesota, USA.

**Keywords:** Pulmonology, Stem cells, Adult stem cells, Hypoxia

## Abstract

Hypoxia is a sentinel feature of idiopathic pulmonary fibrosis (IPF). The IPF microenvironment contains high lactate levels, and hypoxia enhances cellular lactate production. Lactate, acting through the GPR81 lactate receptor, serves as a signal molecule regulating cellular processes. We previously identified intrinsically fibrogenic mesenchymal progenitor cells (MPCs) that drive fibrosis in the lungs of patients with IPF. However, whether hypoxia enhances IPF MPC fibrogenicity is unclear. We hypothesized that hypoxia increases IPF MPC fibrogenicity via lactate and its cognate receptor GPR81. Here we show that hypoxia promotes IPF MPC self-renewal. The mechanism involves hypoxia-mediated enhancement of LDHA function and lactate production and release. Hypoxia also increases HIF1α levels, and this increase in turn augments the expression of GPR81. Exogenous lactate operating through GPR81 promotes IPF MPC self-renewal. IHC analysis of IPF lung tissue demonstrates IPF MPCs expressing GPR81 and hypoxic markers on the periphery of the fibroblastic focus. We show that hypoxia enhances IPF MPC fibrogenicity in vivo. We demonstrate that knockdown of GPR81 inhibits hypoxia-induced IPF MPC self-renewal in vitro and attenuates hypoxia-induced IPF MPC fibrogenicity in vivo. Our data demonstrate that hypoxia creates a feed-forward loop that augments IPF MPC fibrogenicity via the lactate/GPR81/HIF1α pathway.

## Introduction

Idiopathic pulmonary fibrosis (IPF) is a lethal progressive fibrotic lung disease, where the unrelenting scarring of alveolar gas-exchange units results in progressive hypoxia and death by asphyxiation (1–6). Although hypoxia is a sentinel feature of IPF, the precise role that hypoxia plays in disease progression remains incompletely understood. We have previously shown that hypoxia stimulates the proliferation of IPF fibroblasts via upregulation of miR-210, which represses the c-myc inhibitor MNT (7). In addition, we discovered intrinsically fibrogenic mesenchymal progenitor cells (MPCs) in the human IPF lung that serve as a source for IPF fibroblasts (8). IPF MPCs display a distinct transcriptome and a durable fibrogenic phenotype (9–11). Importantly, IPF MPCs produce nonresolving interstitial pulmonary fibrosis when administered to immunodeficient mice, suggesting an important role in IPF fibrotic progression (9). However, whether hypoxia enhances IPF MPC fibrogenicity remains to be determined. Understanding the role of hypoxia in the regulation of IPF MPC fibrogenicity will help elucidate their role in fibrosis progression.

In a hypoxic environment, lactate production increases as the anaerobic cycle is utilized for energy production. While lactate has long been known to play an essential role in cellular metabolism, more recently, lactate has been discovered to serve as a signal molecule regulating a variety of cellular processes associated with cancer progression and cardiovascular disease (12–15). In this regard, the G-protein–coupled receptor 81 (GPR81), also known as hydroxycarboxylic acid receptor 1 (HCAR1), serves as a receptor for lactate (16, 17). GPR81 is upregulated in cancer, where it functions in an autocrine role to promote cancer cell proliferation and in a paracrine role where its presence in DCs prevents the presentation of tumor-specific antigens to immune cells (18, 19). In IPF, lactate levels are elevated and promote myofibroblast differentiation and activation of TGF-β (20–23). Furthermore, prior work has shown that metabolic reprogramming characterized by increased glycolysis is a feature of fibrogenic fibroblasts and that this occurs in the absence of hypoxia, a phenomenon termed the Warburg Effect (24). Since IPF MPCs are intrinsically fibrogenic under normoxic conditions, these findings prompted us to examine: (a) whether there were differences in lactate production between IPF and control MPCs under normoxic conditions and (b) whether hypoxia can enhance lactate production and release and, by operating through the lactate receptor GPR81, further augment IPF MPC fibrogenicity.

Here, we show that the highly cellular region at the periphery of the fibroblastic focus contains IPF MPCs expressing hypoxic markers. We show that, under normoxic conditions, the intrinsically fibrogenic IPF MPCs display increased levels of markers of glycolysis and lactate compared with control MPCs. Importantly, we demonstrate that hypoxia markedly enhances lactate production and release in IPF MPCs. We show that hypoxia increases HIF1α levels. In turn, HIF1α binds to the lactate receptor GPR81 promoter, thereby increasing the expression of GPR81, suggesting that, in response to hypoxic conditions, HIF1α promotes GPR81 expression via increasing GPR81 transcription. We demonstrate that exposure to hypoxic conditions promotes IPF MPC self-renewal. Similar to hypoxia, exogenous administration of lactate promotes IPF MPC self-renewal. Knockdown of GPR81 abrogates hypoxia-induced IPF MPC self-renewal in vitro, and loss of GPR81 function markedly diminishes IPF MPC fibrogenicity in both a humanized mouse model of sustained interstitial fibrosis and in a zebrafish fibrosis model. Our results indicate (a) that cells on the periphery of the fibroblastic focus display markers of hypoxia that codistribute with IPF MPCs and (b) that hypoxia creates a feed-forward loop that enhances IPF MPC fibrogenicity via augmenting lactate production, release, and signaling via the GPR81 lactate receptor. These results identify the lactate/GPR81 axis as a potential therapeutic target to arrest IPF fibrotic progression.

## Results

### The periphery of the IPF fibroblastic focus contains IPF MPCs, which codistribute with markers of hypoxia.

**Figure 1:**
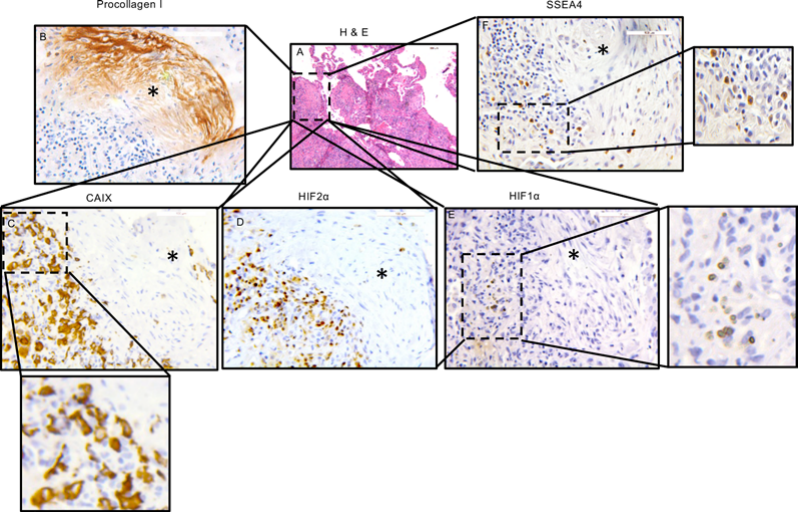
The periphery of the IPF fibroblastic focus contains IPF MPCs, which codistribute with markers of hypoxia. (**A**) IHC analysis was performed on human IPF lung tissue (*n* = 6 IPF patient specimens, 5 sections were imaged in each specimen). H&E staining was used to identify the fibroblastic focus. (**B**–**E**) IHC was performed using antibodies to procollagen to assess collagen synthesis (**B**) and HIF1α, HIF2α, and CAIX as markers of hypoxia (**C**–**E**). (**F**) IHC staining was performed using SSEA4 to identify IPF MPCs. (**A**) Scale bar: 200 μm. (**C**–**F**) Scale bar: 100 μm. Focus cores denoted by asterisks.

We previously discovered that the IPF lung contains intrinsically fibrogenic MPCs that cause nonresolving interstitial lung fibrosis in a humanized mouse xenograft model (9). We found that the IPF fibroblastic focus is a polarized structure with fibrogenic IPF MPCs and their transit-amplifying progeny localizing to a highly cellular region on the periphery of the focus core. The focus core is a more mature region containing noncycling myofibroblasts synthesizing collagen (8–10, 25, 26). Interestingly, studies in cancer suggest that cancer progenitor cells exist in hypoxic niches that support their self-renewal. Therefore, we performed IHC analysis on IPF lung tissue to determine whether cells in the fibroblastic focus display markers of hypoxia. We used HIF1α, HIF2α, and carbonic anhydrase IX (CAIX) as markers of hypoxia (27, 28). Cells in the highly cellular region on the periphery of the focus core stained positive for HIF1α, HIF2α, and CAIX (Figure 1). These results are consistent with our prior results showing expression of the master hypoxamir miR-210 in this region of the fibroblastic focus (7). We also performed IHC on IPF lung tissue using antibodies for SSEA4, a MPC marker, to determine whether IPF MPCs colocalize with hypoxic markers. IPF MPCs were present in a similar distribution with cells expressing HIF1α, HIF2α, and CAIX at the periphery of the fibroblastic focus (Figure 1). Taken together, these findings indicate that the highly cellular region at the periphery of the fibroblastic focus displays markers of hypoxia that codistribute with IPF MPCs.

### Hypoxia promotes IPF MPC self-renewal and enhances lactate production and excretion.

**Figure 2:**
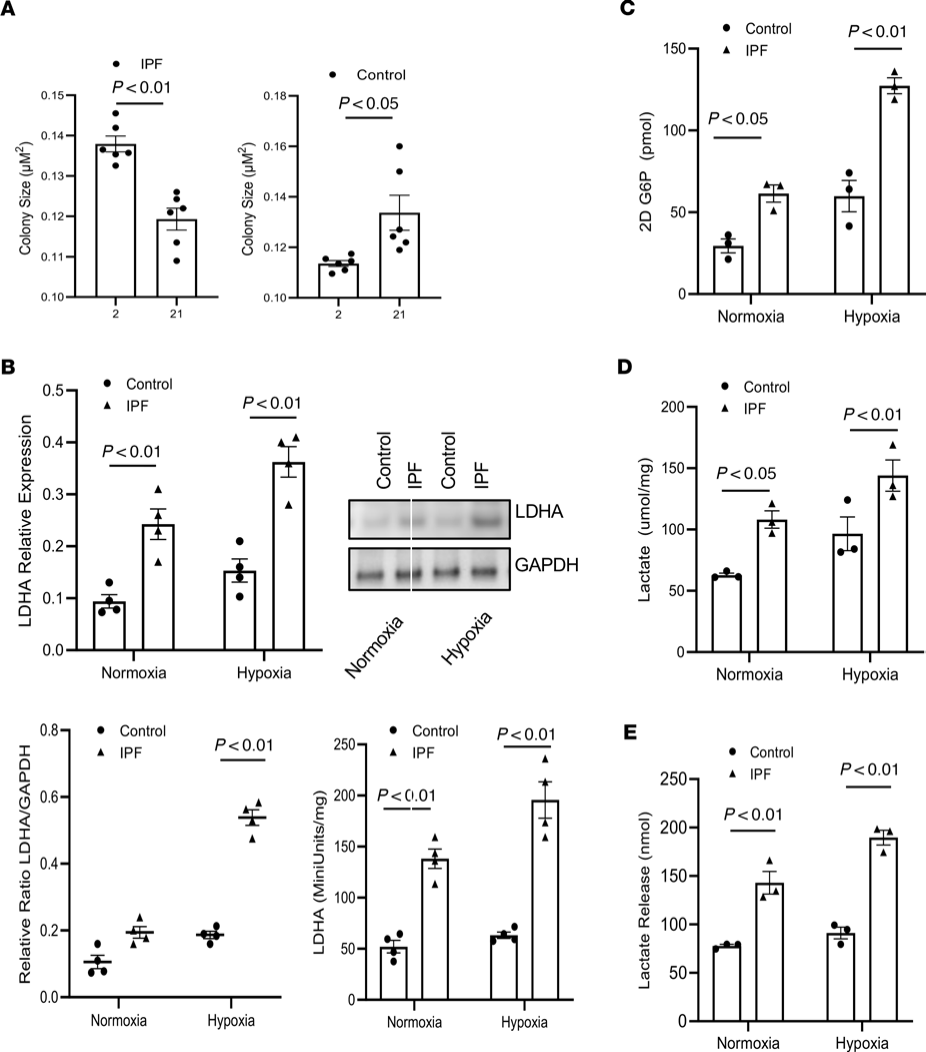
Hypoxia promotes IPF MPC self-renewal and enhances lactate production and excretion. (**A**) IPF (left panel) and control (right panel) MPCs (*n* = 6 each of control and IPF cell lines) were cultured under normoxic (21% O_2_) or hypoxic (2% O_2_) conditions for 7 days. At day 7, self-renewal was quantified. (**B**) IPF and control MPCs were exposed to normoxic or hypoxic conditions. LDHA expression was analyzed by qPCR (left panel) and Western blot (middle left panel). Densitometry values summarizing Western blot data are shown in the middle right panel. GAPDH served as a loading control. LDHA activity was quantified (right panel). *n* = 4, each of control and IPF cell lines. (**C**–**E**) IPF and control MPCs were cultured under normoxic and hypoxic conditions for 24 hours. Glucose uptake (**C**), lactate concentration (**D**), and lactate release (**E**) were quantified. For **C–E**, *n* = 3 each of control and IPF cell lines used. *P* values were determined by 2-tailed Student’s *t* test.

Worsening hypoxia is a prominent clinical feature of IPF fibrotic progression (29). Importantly, hypoxia is a potent stimulus for progenitor cell self-renewal (30, 31). Since IPF MPCs localize to a hypoxic region in the IPF lung, we directly examined the effect of hypoxia on IPF MPC function. Although O_2_ concentrations vary widely in tissues, at the cellular level, physiological O_2_ concentrations are generally in the 1%–11% range (32). With regard to pathological O_2_ concentrations, moderate tissue hypoxia has been defined as ~2.5% (33–35), while O_2_ concentrations in severely hypoxic tissue range from less than 0.1% to 0.5% (33). The level of O_2_ in the normal lung parenchyma has been measured to be 14% (35); however, to our knowledge, there have been no studies that have directly measured the partial pressure of O_2_ in IPF lung tissue. We have previously shown that low O_2_ levels in the range of 3%–5% robustly promote IPF fibroblast proliferation compared with 21% O_2_ (7) and human mesenchymal stromal cells proliferate better at 2% O_2_ compared with normoxia (31). Therefore, we examined the effect of hypoxia on IPF and control MPC self-renewal. Compared with 21% O_2_, 2% O_2_ increased IPF MPC self-renewal (Figure 2A; left panel) but exerted a modest inhibitory effect on control MPC self-renewal (Figure 2A; right panel). Since 2% O_2_ promoted IPF MPC self-renewal and this level of O_2_ tension corresponds to moderate tissue hypoxia, 2% O_2_ concentration was used for the remainder of the experiments unless specified.

Since hypoxia enhanced IPF MPC self-renewal, we next sought to elucidate the mechanism. Prior work indicates that IPF lung tissue contains high lactate levels (20) and that, under normoxic conditions, metabolic reprogramming characterized by increased glycolysis is a feature of fibrogenic fibroblasts (24). Therefore, we first examined whether there were differences in lactate production between IPF and control MPCs in response to normoxic and hypoxic conditions. During glycolysis, lactate dehydrogenase A (LDHA) catalyzes the conversion of pyruvate to lactic acid (13–15). We measured LDHA expression and activity in IPF and control MPCs under normoxic and hypoxic conditions. LDHA mRNA and protein expression and LDHA activity were increased in IPF MPCs compared with control under normoxic conditions, and hypoxia further enhanced LDHA activity in IPF MPCs (Figure 2B). Hypoxia also modestly increased LDHA activity and expression in control MPCs. Since LDHA expression and activity were elevated in IPF MPCs compared with control MPCs, we next examined for evidence of a glycolytic shift in response to hypoxia. We quantified glycolytic flux by measuring glucose uptake and lactate levels in IPF and control MPC cellular lysates as well as lactate excretion by IPF and control MPCs under normoxic and hypoxic conditions. We found that glucose uptake was higher in IPF MPCs compared with control MPCs when cultured under normoxic conditions and that exposure to hypoxic conditions markedly enhanced glucose uptake in IPF MPCs (Figure 2C). Importantly, exposure to hypoxic conditions also greatly enhanced lactate levels in IPF MPCs (Figure 2D). In contrast, hypoxic conditions only modestly increased lactate levels in control MPCs compared with exposure to normoxia. When comparing the effect of exposure to normoxia and hypoxia on lactate levels in IPF and control MPCs, there was a 31% elevation in lactate levels in IPF MPCs versus control MPCs under normoxic conditions. Hypoxia greatly magnified this difference in lactate levels between IPF and control MPCs. Hypoxia increased lactate levels in IPF MPCs 97% compared with control MPCs (Figure 2D). In addition, in response to hypoxia, IPF MPCs also released more lactate compared with control MPCs (Figure 2E). We next examined additional markers of glycolysis to confirm that IPF MPCs undergo a glycolytic shift in response to hypoxia. Expression of both the lactate transporter monocarboxylate transporter 1 (MCT1) and the key glycolytic protein hexokinase II (HKII) was increased in IPF MPCs in response to hypoxia (Supplemental Figure 1; supplemental material available online with this article; https://doi.org/10.1172/jci.insight.163820DS1). These data indicate that IPF MPCs, which are intrinsically fibrogenic, display increased glycolysis and higher levels of lactate compared with control MPCs under normoxic conditions and that hypoxia markedly enhances intracellular lactate levels in IPF MPCs.

### Hypoxia and lactate stimulate IPF MPC self-renewal and migration.

**Figure 3:**
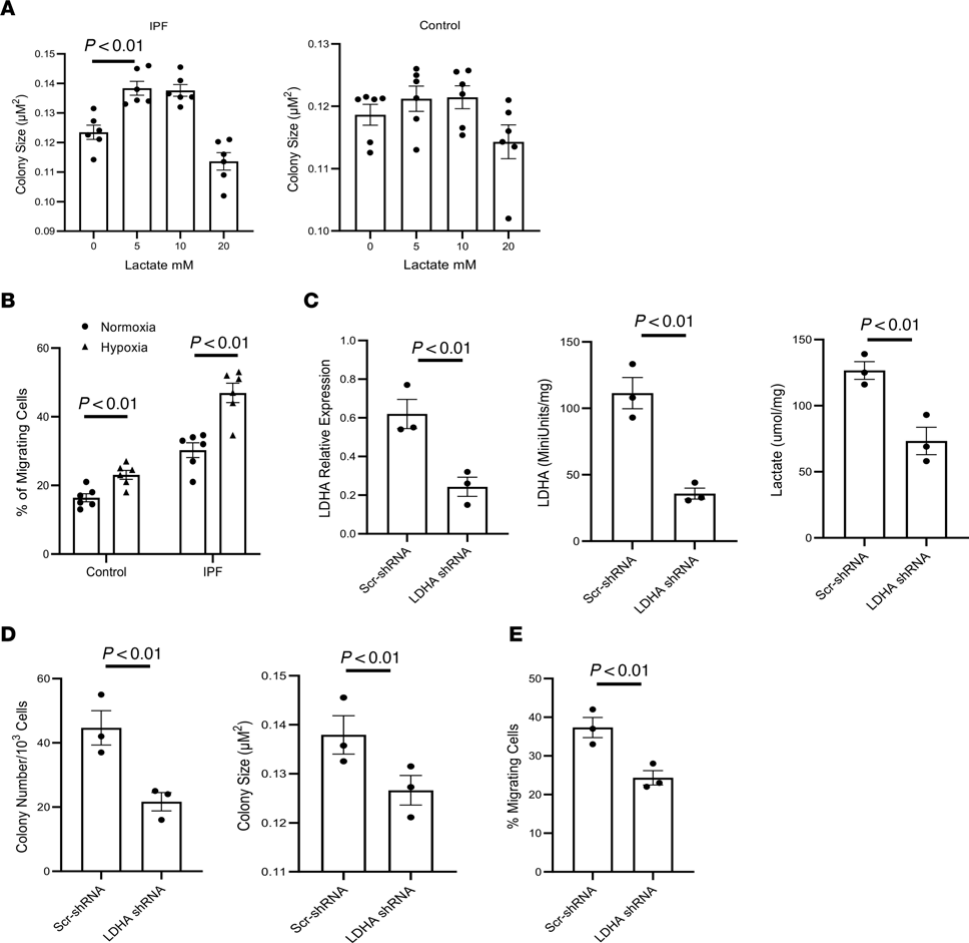
Hypoxia and lactate stimulate IPF MPC self-renewal and migration. (**A**) IPF and control MPCs were treated with the indicated lactate concentrations for 7 days. At day 7, self-renewal was quantified. *n* = 6 each of control and IPF cell lines. (**B**) IPF and control MPCs were seeded in a modified Boyden chamber and cultured under normoxic or hypoxic conditions for 16 hours, after which the number of migrating cells were quantified. *n* = 6 each of control and IPF cell lines. (**C**–**E**) LDHA was knocked down in IPF MPCs using LDHA shRNA (LDHA-shRNA). Scrambled shRNA (Scr-shRNA) served as control. The cells were cultured under hypoxic conditions. LDHA expression was quantified by qPCR (Figure 2C; left panel). LDHA activity was quantified (Figure 2C; middle panel). Lactate levels were quantified in cell lysates (Figure 2C; right panel). (**D**) Self-renewal was assessed in the colony forming assay. (**E**) IPF MPC migration was quantified. For **C**–**E**, *n* = 3 IPF cell lines used. Data are shown as mean ± SEM. *P* values were determined by 2-tailed Student’s *t* test.

Since hypoxia enhances lactate production and release in IPF MPCs, we next examined the effect of exogenous lactate (0, 5, 10, and 20 mM lactate) on IPF MPC self-renewal. Compared with no exogenous lactate, both 5 mM and 10 mM lactate stimulated IPF MPC self-renewal, while IPF MPC self-renewal was slightly lower with 20 mM lactate (Figure 3A; left panel). Exogenous lactate had a minimal effect on control MPC self-renewal (Figure 3A; right panel). These data indicate that both hypoxia and lactate stimulate IPF MPC self-renewal and raise the possibility that hypoxia promotes IPF MPC self-renewal by augmenting lactate production and release. Hypoxia can also promote cell motility (36). Therefore, we examined IPF and control MPC motility in response to normoxic and hypoxic conditions. Hypoxia significantly stimulated IPF MPC motility (Figure 3B). These data indicate that hypoxia stimulates several key MPC functions that could contribute to worsening fibrosis in response to hypoxia.

We next examined the effect of LDHA loss of function on IPF MPC lactate levels and self-renewal in response to hypoxia. Knockdown of LDHA decreased LDHA expression and activity and reduced lactate levels in IPF MPCs in response to hypoxia (Figure 3C). Loss of LDHA function also abrogated the stimulating effect of hypoxia on IPF MPC self-renewal (Figure 3D) and cell motility (Figure 3E). Taken together, these data indicate that hypoxia stimulates LDHA expression and activity and enhances glucose uptake as well as lactate production and release in IPF MPCs; they also suggest that hypoxia may facilitate IPF MPC fibrogenicity by enhancing lactate production.

### IPF MPCs express the lactate receptor GPR81, and IPF MPCs expressing GPR81 are present in a hypoxic niche on the periphery of the fibroblastic focus.

**Figure 4:**
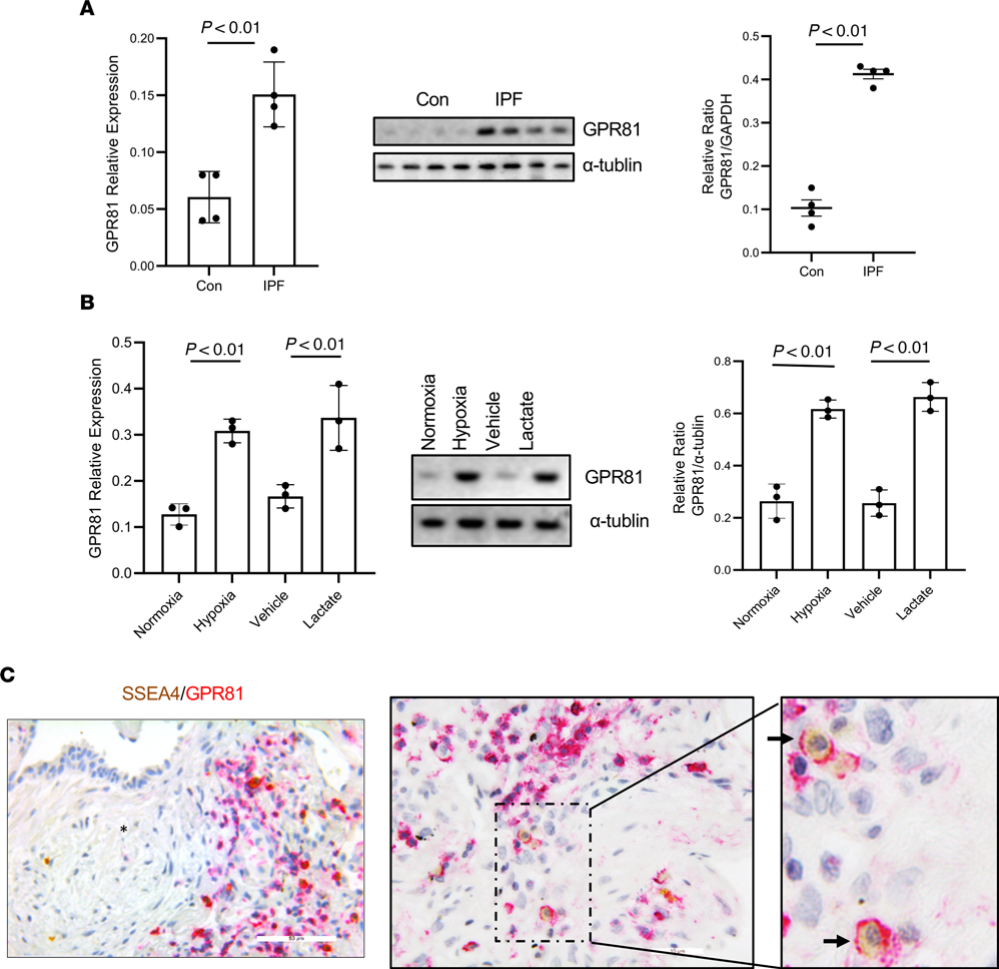
IPF MPCs express the lactate receptor GPR81, and IPF MPCs expressing GPR81 are present in a hypoxic niche on the periphery of the fibroblastic focus. (**A**) GPR81 expression level in IPF and control (Con) MPCs cultured under normoxic conditions were measured with qPCR (left panel) and Western blot (middle panel). Densitometry values summarizing Western blot data are shown in the right panel. α-Tubulin served as a loading control. *n* = 4, each of control and IPF cell lines. (**B**) GPR81 expression levels were quantified in IPF MPCs exposed to normoxic versus hypoxic conditions or 10 mM lactate versus vehicle control by qPCR (left panel) and Western blot (middle panel). Densitometry values summarizing Western blot data are shown in the right panel. α-Tubulin served as a loading control. Three IPF cell lines were used. (**C**) IHC was performed using GPR81 (red) and SSEA4 (brown-yellow) antibodies to assess the distribution of SSEA4 + MPCs expressing GPR81 (*n* = 5, IPF patient specimens; 3 sections were imaged in each specimen). Left panel: SSEA4^+^ cells and GPR81^+^ cells are present on the periphery of the fibroblastic focus). Asterisk indicates myofibroblast core. Arrows denote SSEA4 and GPR81 double positive cells. Middle and right panels show higher-power images of periphery of the fibroblastic focus demonstrating SSEA4^+^GPR81^+^ cells. Scale bar: 50 μm (left); 10 μm (middle). *P* values were determined by 2-tailed Student’s *t* test.

Our in vitro data indicate that hypoxia enhances lactate production and release and exogenous lactate promotes IPF MPC self-renewal. GPR81 is the cognate receptor for lactate, and lactate signaling through GPR81 can regulate a variety of cellular processes (16–19). Therefore, we first analyzed GPR81 expression in IPF and control MPCs. Under normoxic conditions, IPF MPCs expressed higher mRNA and protein levels of GPR81 compared with control (Figure 4A). We next analyzed the effect of hypoxia and exogenous lactate on GRP81 expression in IPF MPCs. Both hypoxia and exogenous lactate markedly enhanced GPR81 expression in IPF MPCs (Figure 4B).

Since both hypoxia and lactate promote IPF MPC self-renewal and since IPF MPCs release lactate and express the lactate receptor GPR81, we performed IHC analysis on IPF lung tissue to determine whether IPF MPCs expressing GPR81 are present on the periphery of the fibroblastic focus. We performed double staining for SSEA4, a MPC marker, and GPR81. SSEA4^+^ MPCs expressing GPR81 were present on the periphery of the fibroblastic focus (Figure 4C and Supplemental Figure 2). These data indicate that the highly cellular region on the periphery of the fibroblastic focus display markers of hypoxia and that this region contains MPCs expressing GPR81. This raises the possibility that hypoxic conditions within this region of the focus promote IPF MPC lactate production, which in turn stimulates IPF MPC self-renewal via GPR81.

### Hypoxia promotes IPF MPC self-renewal and motility via the lactate receptor GPR81.

**Figure 5:**
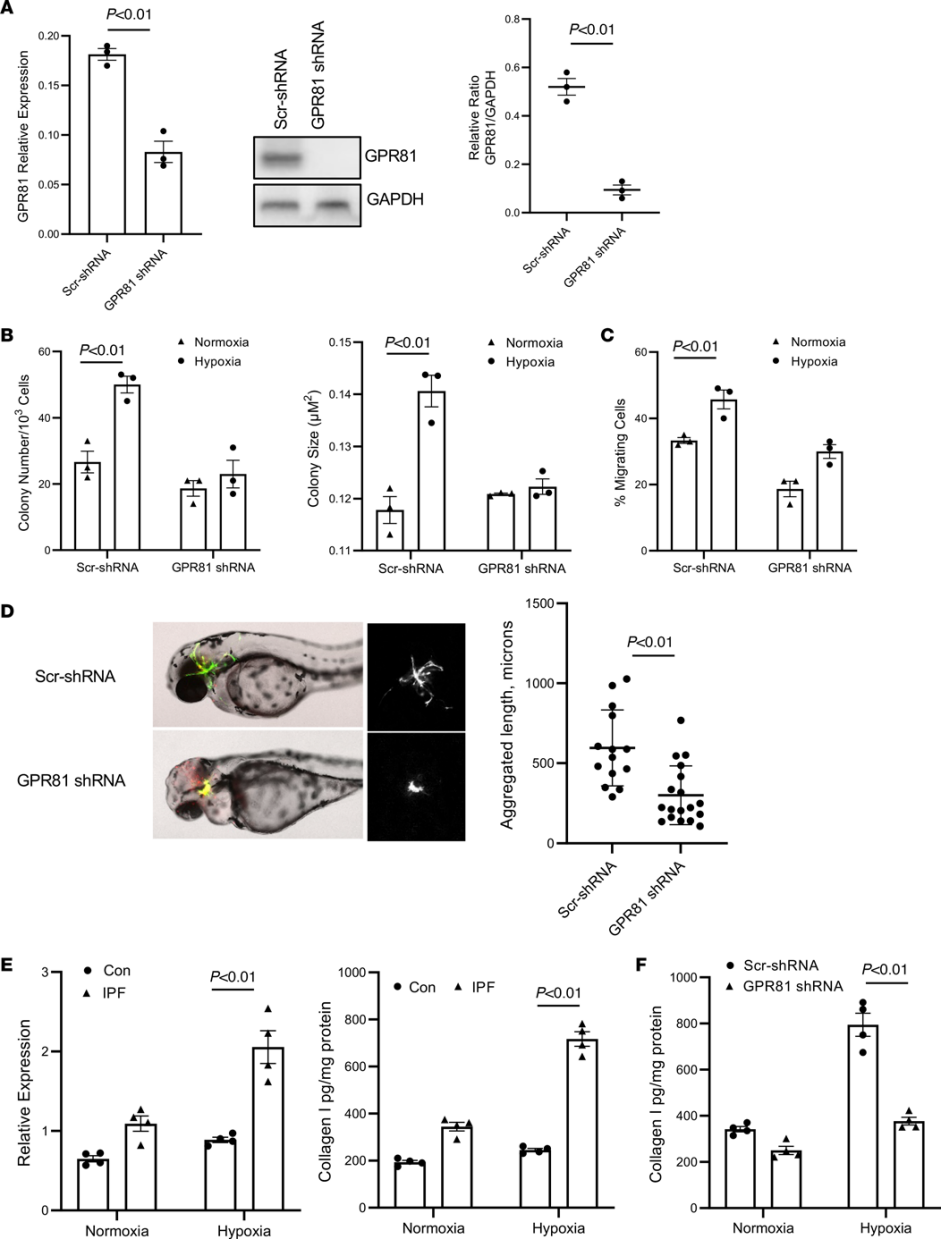
Hypoxia promotes IPF MPC self-renewal and motility via the lactate receptor GPR81. (**A**–**C**) IPF MPCs were transduced with GPR81 shRNA or scrambled shRNA and cultured under hypoxic conditions (2% O_2_). (**A**) GPR81 expression in IPF MPCs were quantified by qPCR (left panel) and Western blot analysis (middle panel). Densitometry values summarizing Western blot data are shown in the right panel. GAPDH served as a loading control. (**B**) IPF MPC self-renewal was assessed in the colony forming assay. (**C**) IPF MPC migration was assessed. Three IPF cell lines were used. Data are shown as mean ± SEM. (**D**) Zebrafish xenograft assay. IPF MPCs transduced with GPR81 or scrambled shRNA (4 independent cells lines) were stained with CFSE, engrafted into zebrafish embryos, and microscopically analyzed in live embryos after 48 hours. Shown are fluorescence (left panels) and bright-field (middle panels) images representative of at least 47 embryos per cell line. The size of the fibrotic reticulum was quantified (right panel). (**E**) IPF and control MPCs were cultured under normoxic and hypoxic conditions. Collagen I mRNA (left panel) and protein (right panel) levels were quantified by qPCR and ELISA. (**F**) IPF MPCs were transduced with GPR81 shRNA or scrambled shRNA and cultured under hypoxic conditions (2% O_2_). Collagen I mRNA (left panel) and protein (right panel) levels were quantified by qPCR and ELISA. For **E** and **F**, *n* = 4 each of control and IPF cell lines used. *P* values were determined by 2-tailed Student’s *t* test.

Physiologically, lactate, acting via its cognate receptor GPR81, serves as a signaling molecule affecting a variety of cellular processes. Pathologically, GPR81 is expressed in multiple cancer cell types, where it promotes tumor cell growth, and silencing of GPR81 dramatically reduces tumor growth and metastasis (18, 37–40). Our data indicate that hypoxia promotes lactate production and release, which support IPF MPC self-renewal, and IPF MPCs express the lactate receptor GPR81. To determine whether hypoxia promotes IPF MPC self-renewal via the lactate/GPR81 axis, we knocked down GPR81 in IPF MPCs. Knockdown of GPR81 decreased GPR81 expression (Figure 5A) and inhibited IPF MPC self-renewal under hypoxic conditions (Figure 5B). In contrast, IPF MPCs transduced with scrambled shRNA demonstrate robust self-renewal in response to hypoxia (Figure 5B).

Since lactate signaling through GPR81 can affect a variety of cellular processes, we also examined the effect on IPF MPC migration. IPF MPCs transduced with scrambled shRNA had higher migration compared with IPF MPCs transduced with GPR81 shRNA (Figure 5C). We previously demonstrated that, when IPF MPCs are xenografted into developing zebrafish embryos, they are motile and invasive forming an extensive fibrotic reticulum, whereas control MPCs form a small mass of nonmotile cells (8, 41). To further examine the ability of GPR81 to regulate IPF MPC motility, we utilized the zebrafish xenograft model. IPF MPCs in which GPR81 had been knocked down and engrafted in zebrafish embryos formed a nonmotile mass of cells. In contrast, IPF MPCs transduced with scrambled shRNA were motile and formed a fibrotic reticulum (Figure 5D).

Prior work indicates that lactate can augment collagen production in fibroblasts (20). Therefore, we also examined the effect of normoxia and hypoxia on collagen synthesis in IPF and control MPCs in vitro. Compared with normoxia, hypoxia augmented collagen I mRNA (Col1A1) and protein expression in control MPCs by 35% and 20%, respectively (Figure 5E; left panel). In contrast, compared with normoxia, hypoxia enhanced collagen I mRNA and protein expression in IPF MPCs by 89% and 109%, respectively (Figure 5E; right panel). Therefore, compared with normoxia, hypoxia increased collagen I production to a greater extent in IPF MPCs compared with control MPCs. We also examined collagen I production in IPF MPCs transduced with GPR81 shRNA and scrambled shRNA and exposed to hypoxia. Knockdown of GPR81 decreased collagen I levels by 53% compared with scrambled control (Figure 5F). Taken together, our data support the concept that hypoxia-induced IPF MPC self-renewal, motility, and production of collagen are mediated via the lactate/GPR81 axis.

### The GPR81/PKA-CREB signal pathway regulates IPF MPC self-renewal.

**Figure 6:**
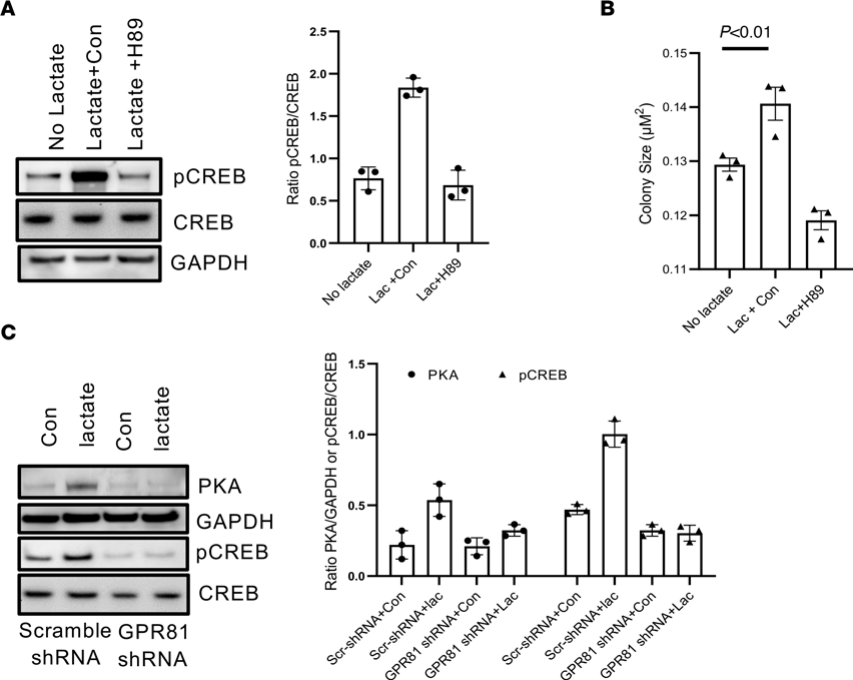
The GPR81/PKA-CREB signal pathway regulates IPF MPC self-renewal. (**A** and **B**) IPF MPCs were cultured in lactate (5 mM) + PKA inhibitor H89 (10 μM) or lactate + vehicle control (Con). Cells cultured in the absence of lactate served as an additional control. Phosphorylated CREB (pCREB) expression was quantified by Western blot analysis (left panel). Total CREB (CREB) and GAPDH served as a loading controls. Densitometry values summarizing Western blot data are shown in the right panel (*n* = 3 cell lines tested). IPF MPC self-renewal was assessed in the colony forming assay (*n* = 6 IPF cell lines). (**C**) PKA and pCREB expression (pCREB ser133) were quantified in IPF MPCs transduced with GPR81 shRNA or scrambled shRNA and cultured in the presence or absence of lactate (5 mM). PKA and pCREB levels were quantified by Western blot analysis (left panel). GAPDH and total CREB served as loading controls. Densitometry values summarizing Western blot data are shown in the right panel. *n* = 3 IPF cell lines examined. *P* values were determined by 2-tailed Student’s *t* test.

GPCRs function via the cAMP/protein kinase A (PKA) pathway and target the cAMP response element-binding protein (CREB) to affect cell function. To begin to assess the pathways downstream of GPR81 that affect IPF MPC self-renewal, we quantified PKA and phosphorylated CREB (pCREB ser133) levels in IPF MPCs in response to exogenous lactate. Lactate increased both PKA and pCREB levels (Figure 6, A and C). Furthermore, the PKA inhibitor H89 inhibited both the lactate-mediated increase in pCREB levels (Figure 6A) and IPF MPC self-renewal (Figure 6B). We also analyzed the effect of exogenous lactate on PKA and pCREB levels in IPF MPCs in which GPR81 had been knocked down. In response to exogenous lactate treatment, PKA and pCREB failed to increase in IPF MPCs transduced with GPR81 shRNA (Figure 6C). In contrast, in response to lactate treatment, both PKA and pCREB levels increased in IPF MPCs transduced with scrambled shRNA. These data support the concept that the mechanism by which lactate signaling through the lactate receptor GPR81 promotes IPF MPC self-renewal involves the cAMP/PKA/CREB pathway.

### Hypoxia promotes GPR81 expression in IPF MPCS via HIF1α.

**Figure 7:**
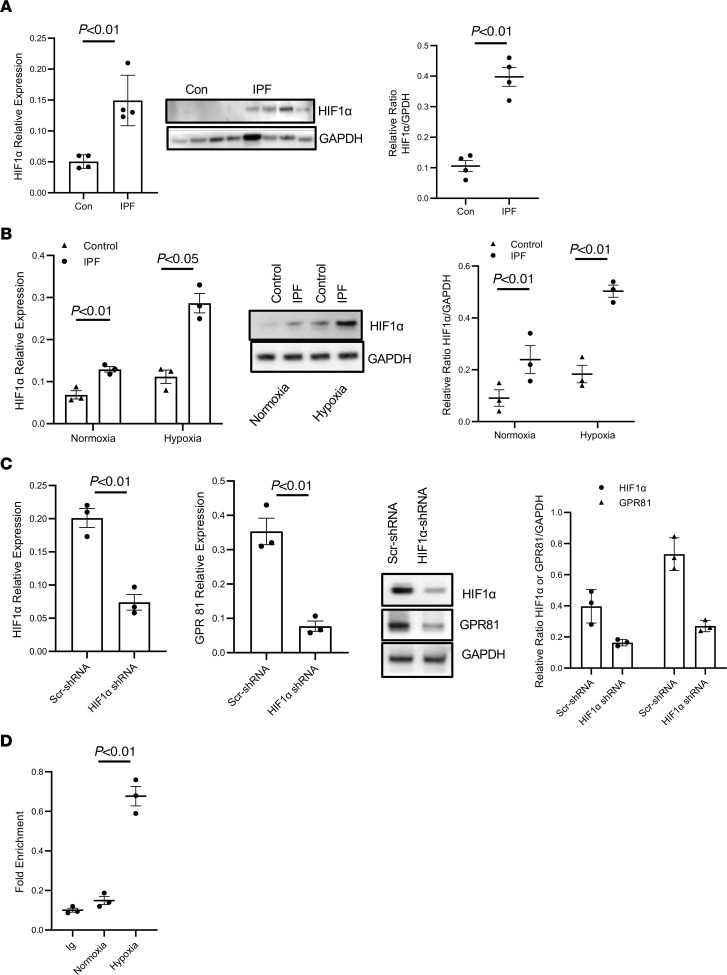
Hypoxia promotes GPR81 expression in IPF MPCS via HIF1α. (**A**) HIF1α expression was quantified in IPF and control MPCs cultured under normoxic conditions by qPCR (left panel) and Western blot (middle panel). Densitometry values summarizing Western blot data are shown in the right panel. GAPDH served as a loading control. *n* = 4, each of control and IPF cell lines. (**B**) HIF1α expression was quantified in IPF and control MPCs exposed to normoxic or hypoxic conditions by qPCR (left panel) and Western blot analysis (middle panel). Densitometry values summarizing Western blot data are shown in the right panel. GAPDH served as a loading control. *n* = 3, each of control and IPF cell lines used. (**C**) IPF MPCs were transduced with HIF1α or scrambled shRNA, and the cells were cultured under hypoxic conditions. HIF1α and GPR81 expression levels were quantified by qPCR (left panel) and Western blot analysis (middle panel). Densitometry values summarizing Western blot data are shown in the right panel. GAPDH served as a loading control. Three IPF cell lines were used. (**D**) ChIP assay. IPF MPCs were cultured under normoxic or hypoxic conditions. HIF1α was immunoprecipitated from nuclear fractions using HIF1α antibody, and qPCR for GPR81 was performed. Immunoprecipitation using isotype antibody (IgG) served as control. Three IPF cell lines were used. Data are shown as mean ± SEM. *P* values were determined by 2-tailed Student’s *t* test.

The HIF1α transcription factor is a major regulator of protein expression in response to hypoxia (42, 43). Since IPF MPC GPR81 expression increases in response to hypoxia, we sought to determine whether this hypoxia-induced expression of GPR81 is mediated via HIF1α. We first examined HIF1α expression in IPF and control MPCs under normoxic and hypoxic conditions. Under normoxic conditions, IPF MPCs expressed higher levels of HIF1α compared with control MPCs (Figure 7A). Importantly, exposure to hypoxia markedly increased HIF1α levels in IPF MPCs by 189% compared with control MPCs. These data indicate that exposure of IPF MPCs to hypoxia magnifies the elevation of HIF1α levels to a much greater extent compared with control MPCs (Figure 7B). To determine whether hypoxia-induced GPR81 expression is regulated by HIF1α, HIF1α was knocked down in IPF MPCs. The transduced cells were then exposed to hypoxic conditions. Knockdown of HIF1α inhibited the ability of hypoxia to induce HIF1α expression (Figure 7C). Importantly, GPR81 mRNA and protein expression were decreased in IPF MPCs in which HIF1α was knocked down compared with cells transduced with scrambled shRNA (Figure 7C). To determine whether HIF1α directly targets the GPR81 gene, we performed ChIP analysis. IPF MPCs were exposed to hypoxia, HIF1α was immunoprecipitated, and PCR for GPR81 was performed. ChIP analysis indicated that HIF1α directly interacted with the GPR81 promoter (Figure 7D), suggesting that, in response to hypoxic conditions, HIF1α promotes GPR81 expression via increasing GPR81 transcription.

### Hypoxia enhances IPF MPC-mediated fibrogenicity in vivo via GPR81.

**Figure 8:**
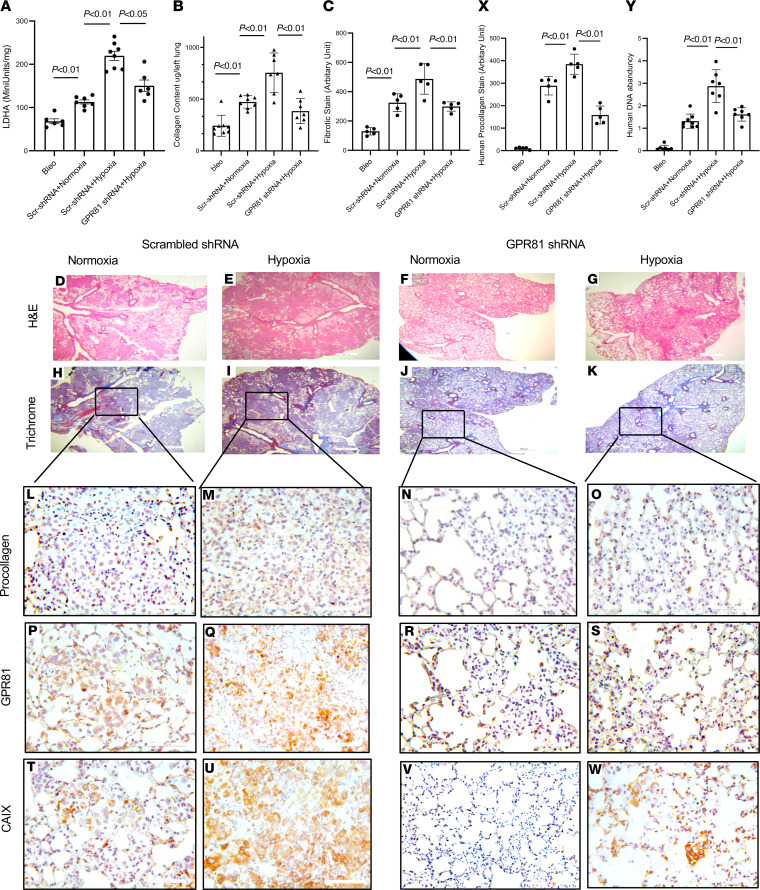
Hypoxia enhances IPF MPC–mediated fibrogenicity in vivo via GPR81. NSG mice were treated with i.t. bleomycin (1.25 U/kg). Two weeks later, the mice received IPF MPCs transduced with either GPR81 shRNA or scrambled shRNA via tail vein injection (1 ***×*** 10^6^ cells/100 μL); 10 mice/group. The mice were maintained under normoxic or hypoxic (10% O_2_) conditions for the duration of the experiment. Lungs were harvested 4 weeks after cell administration. (**A**) LDHA activity. (**B**) Collagen content was quantified in left lungs by Sircol assay (left panel). (**C**) Semiquantitative analysis of lung collagen deposition was performed by trichrome staining. Three sections from each animal were screened. Three trichrome stained images randomly selected from each section were used for quantification. Scale bar: 500 μm. The blue regions (fibrotic stain) were defined and quantified with ImageJ (NIH); see images **H**–**K**. (**D**–**W**) Serial 4 μm sections of right lung tissue from mice receiving IPF MPCs transduced with scrambled-shRNA or GPR81 shRNA (**D**–**K**, scale bar: 200 μm; **L**–**W**, scale bar: 50 μm). Representative H&E and trichrome stains assessing fibrosis and collagen deposition, respectively (**D**–**K**). IHC using an antibody recognizing human procollagen to identify human cells and assess collagen synthesis (**L**–**O**); an anti–human GPR81 antibody to determine the distribution of GPR81 expressing cells (**P**–**S**); an anti–human CAIX antibody to determine the distribution of CAIX expressing cells (**T**–**W**). (**X**) Semiquantitative analysis of human procollagen I staining in mouse lungs using ImageJ. (**Y**) Human IPF cell numbers were quantified by qPCR. *P* values were determined by 1-way ANOVA.

Our in vitro studies indicate that hypoxia enhances IPF MPC lactate production and release and promotes IPF MPC self-renewal and motility via the lactate/GPR81 axis. We next sought to determine the effects of hypoxia on IPF MPC fibrogenicity in vivo and the role of GPR81 in this process. To do this, we utilized our humanized mouse xenograft model. In this system, administration of IPF MPCs to immunocompromised NOD/SCID/IL2rγ/B2M (NSG) mice pretreated with intratracheal bleomycin results in nonresolving interstitial lung fibrosis (9). To initiate an experiment, NSG mice were first treated with low-dose intratracheal bleomycin. Two weeks later, they received IPF MPCs transduced with scrambled shRNA or GPR81 shRNA via tail vein. The mice were maintained under normoxic or hypoxic (10% O_2_) conditions (10% was the lowest O_2_ concentration permitted by the University of Minnesota IACUC). Lungs were harvested 4 weeks later. LDHA activity as a surrogate marker of lactate production was quantified. LDHA activity in the lungs of mice transduced with scrambled shRNA and exposed to hypoxia was increased compared with mice exposed to normoxia (Figure 8A; left panel). Interestingly, LDHA activity was modestly lower in hypoxic mice receiving IPF MPCs transduced with GPR81 shRNA compared with scrambled shRNA (Figure 8A; right panel). These data suggest that lactate production is increased in the lungs of mice exposed to hypoxia and that GPR81 knockdown had only modest effects on lactate production in mice exposed to hypoxia.

We next quantified the level of fibrosis in mice treated with IPF MPCs and exposed to normoxic and hypoxic conditions. We found that the collagen content and lung fibrosis score in mice receiving IPF MPCs transduced with scrambled shRNA and exposed to hypoxia were increased compared with mice receiving IPF MPCs transduced with scrambled shRNA and maintained under normoxic conditions (Figure 8, B and C; left hand panels). This indicates that hypoxia augments IPF MPC–mediated interstitial fibrosis. Consistent with this, H& E and trichrome staining showed more extensive regions of fibrosis, and IHC analysis demonstrated more regions with increased collagen deposition containing human cells that expressed procollagen in mice that received IPF MPCs transduced with scrambled shRNA and exposed to hypoxia (Figure 8, E, I, and M) compared with their counterparts exposed to normoxia (Figure 8, D, H, and L). Of note, GPR81- and CAIX-expressing cells were more prevalent in the fibrotic regions of mice exposed to hypoxic conditions and receiving IPF MPCs transduced with scrambled shRNA (Figure 8, Q and U) compared with those exposed to normoxia (Figure 8, P and T). The lungs of these mice also had increased collagen content and a higher lung fibrosis score by Trichrome staining compared with mice treated with bleomycin only (Figure 8, B and C). These data are consistent with our prior studies demonstrating the ability of IPF MPCs to cause nonresolving interstitial fibrosis, while their control counterparts do not (9).

We next compared the fibrogenicity of IPF MPCs transduced with GPR81 to those transduced with scrambled shRNA in the humanized mouse model under hypoxic conditions. The collagen content and lung fibrosis score were significantly lower in mice receiving IPF MPCs in which GPR81 had been knocked down and exposed to hypoxia compared with mice receiving IPF MPCs transduced with scrambled shRNA and exposed to hypoxia (Figure 8, B and C; right panels). Knockdown of GPR81 reduced IPF MPC fibrogenicity in response to hypoxia by 42% (collagen content 657.19 ± 34.18 μg/left lung versus 377.78 ± 28.67 μg in left lung). Consistent with this, there were more extensive regions of lung fibrosis in mice exposed to hypoxia and receiving IPF MPCs transduced with scrambled shRNA (Figure 8, E and I) compared with mice receiving IPF MPCs in which GPR81 had been knocked down (Figure 8, G and K). IHC analysis of lung tissue from mice exposed to hypoxia and receiving IPF MPCs transduced with scrambled shRNA contained more numerous human cells expressing procollagen, GPR81, and CAIX in fibrotic regions (Figure 8, M, Q, and U) compared with the lungs of mice receiving IPF MPCs in which GPR81 had been knocked down (Figure 8, O, S, and W). This is consistent with our in vitro data demonstrating that knockdown of GPR81 inhibits hypoxia-induced IPF MPC self-renewal. In addition, the degree of fibrosis in mice receiving IPF MPCs in which GPR81 had been knocked down and exposed to normoxia (Figure 8, F, J, N, R, and V) were not significantly different from mice receiving IPF MPCs in which GPR81 had been knocked down and exposed to hypoxia. Of note the antibody used to detect human cells, human procollagen I, is specific for human cells and does not stain mouse cells. Semiquantitative analysis of human procollagen I staining in mice exposed to hypoxic conditions demonstrated that knockdown of GPR81 in IPF MPCs decreased human procollagen I staining in the mouse lungs by 40% compared with mice exposed to hypoxia and receiving IPF MPCs transduced with scrambled control (Figure 8X). These data are in accord with the interpretation that there are fewer human cells producing human procollagen I in mice receiving IPF MPCs transduced with GPR81 shRNA compared with control.

To quantify the number of human cells in mice receiving IPF MPCs transduced with GPR81 shRNA or scrambled shRNA, at the time of engraftment and at lung tissue harvest, we used a real-time PCR method shown to be sensitive for the detection and quantification of human cells in mice (44). The lungs of mice receiving IPF MPCs transduced with scrambled shRNA and exposed to hypoxic conditions for 4 weeks contained higher levels of human DNA compared with mice that received IPF MPCs in which GPR81 was knocked down and exposed to hypoxia (Figure 8Y). Furthermore, the level of human DNA found in the lungs of mice receiving IPF MPCs transduced with GPR81 shRNA and exposed to hypoxia was roughly equivalent to that seen in mice receiving IPF MPCs transduced with scrambled shRNA and exposed to normoxic conditions. Taken together, these data indicate that, under hypoxic conditions, there are more human cells producing collagen in the lungs of mice receiving IPF MPCs transduced with scrambled shRNA compared with mice receiving IPF MPCs transduced with GPR81 shRNA. These data demonstrate that hypoxia augmented IPF MPC fibrogenicity in vivo and that knockdown of GPR81 diminished this effect. This supports the concept that hypoxia enhances IPF MPC–mediated fibrogenesis via the lactate/GPR81 axis.

When we analyzed the engraftment of human IPF MPCs transduced with scrambled and GPR81 shRNA in the lungs of the NSG mice using real-time PCR, we found no significant differences excluding engraftment differences as the explanation for our results (Supplemental Figure 3). In addition, we examined the effect of exposure of IPF MPCs transduced with GPR81 or scrambled control to 10% O_2_ (the concentration of O_2_ used in the in vivo studies) on induction of apoptosis in vitro by quantifying caspase 3 levels. No significant change in caspase 3 levels was found (Supplemental Figure 4), indicating that in vitro exposure to 10% O_2_ concentration does not promote apoptosis of IPF MPCs in which GPR81 expression has been knocked down. Taken together, these data indicate that both groups received equal numbers of cells and that the difference in fibrosis between the groups resulted from different biological properties of scrambled shRNA and GPR81 shRNA cells.

We also examined the effects of exposure of 10% O_2_ on mouse lung tissue hypoxia using Hydroxyprobe. For this experiment, the mice were maintained under hypoxic (10% O_2_) or normoxic conditions for 48 hours. The lungs were harvested and stained with Hydroxyprobe (45). Compared with mice maintained under normoxic conditions, the lungs of mice exposed to hypoxic conditions demonstrated extensive Hydroxyprobe staining (Supplemental Figure 5). This indicates that exposure to 10% O_2_ indeed created a hypoxic lung environment.

## Discussion

IPF is a chronic and ultimately fatal disease characterized by a progressive decline in lung function (1, 46). In IPF, there is progressive spread of fibrosis from scarred alveolar units into contiguous alveoli creating expanding regions of fibrotic tissue that obliterate the gas exchange apparatus and impair oxygenation (47). This progressive fibrosis clinically correlates with worsening hypoxia (29). Although hypoxia is such a prominent clinical feature of IPF, few mechanistic studies have explored the role of hypoxia as a driver of IPF fibrotic progression (48–51). In support of this concept, there is considerable experimental evidence that tissue hypoxia leads to fibrosis (52–60). Recently, we discovered that hypoxia stimulates IPF fibroblast proliferation by a mechanism involving HIF2α-mediated upregulation of miR-210 (7). These results support the concept that a pathologic circuit operates in the IPF lung, in which hypoxia promotes proliferation of fibroblasts, which in turn worsens hypoxia. Interdicting hypoxia-mediated feed-forward loops could provide a means to arrest fibrotic progression.

Hypoxic regions within tissue microenvironments function as critical niches that support stem/progenitor cell self-renewal (30, 31, 61). We have discovered that the IPF fibroblastic focus is a polarized structure containing proliferating fibrogenic MPCs that serve as a source of IPF fibroblasts located within an active fibrotic front and noncycling, collagen-synthesizing myofibroblasts within a core region (8, 9). Our immunohistochemical (IHC) studies indicate that the highly cellular region at the periphery of the fibroblastic focus displays markers of hypoxia which codistribute with IPF MPCs. We demonstrate that cells staining positive for CAIX, HIF1α and HIF2, which are upregulated by hypoxia and serve as markers of tissue hypoxia, are present around the fibroblastic focus (27, 28). These data are consistent with our previous studies in which we demonstrated that miR-210, a master hypoxamir whose expression is upregulated by hypoxia, is highly expressed in cells at the periphery of the fibroblastic focus (7). We hypothesized that this region of the fibroblastic focus may support IPF MPC function, including self-renewal. Here, we show that hypoxia promotes IPF MPC self-renewal. Mechanistically, we found that hypoxia upregulates the expression of LDHA, enhancing lactate production and release in IPF MPCs. We demonstrate that exogenous lactate also promotes self-renewal of IPF MPCs. We show that hypoxia increases HIF1α levels. In turn, HIF1α binds to the lactate receptor GPR81 promoter, thereby increasing the expression of GPR81. This suggests that, in response to hypoxic conditions, HIF1α promotes GPR81 expression via increasing GPR81 transcription. Importantly, we demonstrate that knockdown of GPR81 inhibits hypoxia-mediated IPF MPC self-renewal in vitro. Using a mouse xenograft model of sustained interstitial lung fibrosis, we show that hypoxia drove lung fibrosis and that loss of GPR81 function abrogated the effect of hypoxia on progression of lung fibrosis. Together, our data support the concept that hypoxia, acting via the lactate/GPR81/HIF1α pathway, enhances IPF MPC fibrogenicity driving fibrotic progression.

We have previously shown that IPF MPCs are intrinsically fibrogenic when cultured under normoxic conditions (8, 9). Prior studies have determined that, under normoxic conditions, metabolic reprogramming characterized by increased glycolysis is a feature of fibrogenic fibroblasts (24). Consistent with this, here we show that, when cultured under normoxic conditions, both markers of glycolysis and lactate production were elevated in IPF MPCs compared with control MPCs. Under hypoxic conditions, cells produce energy by increasing the rate of glycolysis (62). LDHA is a critical enzyme that catalyzes the final step of glycolysis in which pyruvate is concerted to lactate (13–15). Consistent with this, we show exposure of IPF MPCs to hypoxia augmented LDHA expression and activity, as well as markers of glycolysis, and enhanced lactate production and release. Furthermore, hypoxia greatly magnified the differences in lactate levels between IPF and control MPCs. Importantly, when compared with exposure to normoxia, hypoxic conditions modestly increased lactate levels by 21% in control MPCs. However, exposure to hypoxic conditions markedly augmented lactate levels in IPF MPCs, increasing lactate levels by 65% compared with normoxia. Taken together, these data demonstrate that glycolysis and lactate production are higher in intrinsically fibrogenic IPF MPCs compared with control cells when cultured under normoxic conditions and that hypoxia creates a feed-forward loop that greatly enhances the increase in intracellular lactate levels in IPF MPCs (Supplemental Figure 6).

Prior work has demonstrated that lactate levels are elevated in IPF and that elevated lactate levels may be a driver of fibrosis progression (20–23). While the precise mechanisms by which elevated lactate may facilitate fibrotic progression remain to be elucidated, one study found that increased lactate levels activate TGF-β, which induces myofibroblast differentiation and production of collagen (20). Interestingly, in cancer, elevation of lactate levels promotes cancer cell growth, serves as a biomarker for cancer progression, and is being explored as a therapeutic target (14, 18, 19, 63–65). In our study, we demonstrate that hypoxia and lactate promote IPF MPC self-renewal. Knockdown of LDHA blocks the ability of hypoxia to induce IPF MPC self-renewal, supporting the concept that hypoxia generates increased lactate levels via LDHA, which supports pathologic IPF MPC function. Since IPF MPCs serve as a source of IPF fibroblasts, we suggest that the hypoxia-driven enhancement in lactate levels promotes IPF MPC self-renewal and expansion of the pathologic fibroblast population, which serve as a critical drivers of fibrosis.

Like IPF, the tumor microenvironment is characterized by low O_2_ and elevated lactate levels (66). Studies in cancer support the idea that tumor cells adapt to this low O_2_, high lactate microenvironment by increasing the expression of the lactate receptor GPR81 (18, 19). In cancer, lactate produced by tumor cells activates GPR81 and supports tumor growth and metastasis (18). Similar to cancer, our IHC studies suggest that GPR81-expressing MPCs are located in a hypoxic region on the periphery of the IPF fibroblastic focus. This raises the possibility that the IPF MPCs have adapted to this hypoxic region by increasing GPR81 expression. This concept is supported by our in vitro studies showing that, in addition to increasing lactate production and release, hypoxia upregulates GPR81 expression, and knockdown of GPR81 blocks hypoxia-mediated IPF MPC self-renewal and motility. Many of the effects of hypoxia are mediated through the HIF1α transcription factor (67–69). We show that hypoxia increases GPR1 expression via HIF1α and that HIF1α binds to the GPR81 promoter, suggesting that HIF1α directly upregulates GPR81 expression. Taken together, our data provide a paradigm of IPF fibrotic progression, where a low O_2_, high lactate microenvironment on the periphery of the fibroblastic focus serves as a fibroproliferative niche by supporting IPF MPC function and expansion of the IPF fibroblast population.

This paradigm is supported by our in vivo studies. We have developed a mouse xenograft model where administration of human IPF MPCs in immunodeficient mice pretreated with a low dose of i.t. bleomycin creates sustained interstitial lung fibrosis. Here, we show that, compared with mice treated with IPF MPCs and maintained under normoxic conditions, mice treated with IPF MPCs and maintained under hypoxic conditions have more extensive fibrosis. Importantly, we go on to demonstrate that knockdown of GPR81 in IPF MPCs blocks this increase in lung fibrosis in response to hypoxic conditions. Our studies to quantify the number of human cells in the lungs of mice exposed to hypoxia indicate that, in mice receiving IPF MPCs in which GPR81 has been knocked down, fewer human cells expressing procollagen I are present compared with mice receiving IPF MPCs transduced with scrambled shRNA. Our in vitro studies indicate that 10% O_2_ does not significantly increase IPF MPC apoptosis in GPR81-knockdown cells. However, our in vitro data indicate that hypoxia enhances self-renewal and collagen production in IPF MPCs and that knockdown of GPR81 decreases both IPF MPC self-renewal and collagen I production. Taken together, these data suggest that the reduced fibrosis in mice receiving IPF MPCs in which GPR81 has been knocked down is due to decreased proliferation of IPF cells in the mouse lungs or decreased collagen production, or a combination of both.

In summary, these findings indicate that the highly cellular region at the periphery of the fibroblastic focus displays markers of hypoxia that codistribute with IPF MPCs. Our data demonstrate that hypoxia creates a feed-forward loop that further enhances IPF MPC fibrogenicity via the lactate/GPR81/HIF1α pathway. The lactate/GPR81 axis may prove to be an important therapeutic target for IPF.

## Methods

### Primary mesenchymal cell lines.

Six primary lung mesenchymal cell lines were established from 6 individual patients (3 male and 3 female; average age, 63), fulfilling diagnostic criteria for IPF including a pathological diagnosis of usual interstitial pneumonia (1) at the time of lung transplantation. Control MPCs were derived from lung tissue (2 male and 4 female; average age, 57) obtained by video-assisted thoracoscopic surgery (VATS) biopsy or lobectomy and uninvolved from the primary disease process (adenocarcinoma, *n* = 1; carcinoid tumor, *n* = 1; synovial sarcoma, *n* = 1; leiomyosarcoma, *n* = 1; bronchiectasis, *n* = 1; and normal lung tissue, *n* = 1). Cell lines were derived from lungs, characterized as mesenchymal cells, and cultivated as previously described (70).

### Isolation of MPCs.

For isolation of IPF and control MPCs, primary IPF mesenchymal cells were labeled with mouse anti–human SSEA4 antibody conjugated to Alexa Fluor 647 (clone MC-813-70; catalog 560796; BD Biosciences) and mouse anti–human CD44 conjugated to FITC (clone IM7; catalog 103006; BioLegend). Cells were sorted on a FACS Aria Cell Sorter (BD Biosciences). Cells that were SSEA4^+^ and CD44^+^ (relative to mouse IgG3 κ isotype control conjugated to Alexa Fluor 647 and mouse IgM κ isotype control conjugated to FITC, respectively) (clone J606, catalog 560803 [BD Biosciences] and catalog 402207 [BioLegend]) were collected. To generate sufficient numbers of MPCs for the in vivo mouse studies, SSEA4^+^CD44^+^ cells were expanded by culture in DMEM + 10% FCS for 7 days prior to use. The resulting MPC cultures were reanalyzed for SSEA4 expression by FACS analysis and for colony formation in vitro. In total, 97% of day 7 MPCs were SSEA4^+^ and formed colonies in methylcellulose indicating retention of progenitor self-renewal properties during in vitro expansion.

### Hypoxia and lactate treatment.

IPF or control MPCs were cultured under hypoxic (2% O_2_) or normoxic (21% O_2_) conditions for the duration of the experiment. For lactate stimulation, lactate (MilliporeSigma) in the indicated amounts was added to the media. Cells were cultured for 24 hours for most experiments, except where indicated.

### Glucose uptake and lactate assay.

To analyze glucose uptake in IPF MPCs in response to hypoxia, we utilized a glucose uptake assay kit (Abcam). Briefly, IPF MPCs were cultured in RPMI-1640 for 18 hours and were then washed twice with PBS, followed by incubation for 30 minutes in basic RPMI-1640 (no glucose; Thermo Fisher Scientific). The cells were next cultured in Krebs-Ringer-Phosphate-Hepes (KRPH) buffer for 40 minutes, followed by incubation with the glucose analog 2-deoxyglucose (2-DG) for 20 minutes. Following steps per the manufacturer’s instructions, 2-deoxy-D-glucose-6-phosphate (2DG6P) levels were quantified at 420 nm with a SpectraMax M3 microplate reader (Molecular Devices). Lactate levels in IPF MPC lysates and conditioned medium were quantified using a lactate assay kit, per the manufacturer’s instruction (Abcam).

### Self-renewal assay.

Single-cell suspensions of IPF MPCs were incorporated into methylcellulose gels (Stemcell Technologies) and were maintained in MSC SFM CTS (Thermo Fisher Scientific) for 1 week under hypoxic (2% O_2_) or normoxic (21% O_2_) conditions. Enumeration of colonies was performed microscopically, and colony size was quantified by ImageJ (NIH). In some self-renewal assays, the cells were treated with the indicated concentrations of lactate (MilliporeSigma).

### Migration assay.

The effect of hypoxia or lactate (MilliporeSigma) on the migration of IPF and control MPCs was examined using the QCM Chemotaxis Cell Migration Assay Kit following the manufacturer’s instructions (EMD Millipore). Briefly, 2 × 10^4^ IPF MPCs were added to the top of a filter insert (8 μm pore size) containing 100 μL of serum free DMEM in a 96-well plate. A total of 150 μL of DMEM was added to the lower chamber, and the cells were allowed to migrate for 16 hours under hypoxic (2% O_2_) or normoxic conditions (21% O_2_). The number of migrating cells were quantified using CyQuant GR dye and a fluorescence plate reader (SpectraMax M3). Alternatively, in some experiments, DMEM containing lactate was added to the lower well and migration was assessed.

### Quantitative PCR.

LDHA, GPR81, and HIF1α gene expression was conducted by quantitative PCR (qPCR) as previously described (8, 9). Total RNA was isolated and reverse transcribed using a Taqman Reverse Transcriptase Reagent Kit (Roche) and primed with random hexamers. Primer sequences were selected using NCBI Primer-BLAST. qPCR was performed using a LightCycler FastStart DNA Master^PLUS^ SYBR Green I Kit (Roche). Primer sequences were as follows: LDHA Forward: 5′-GCCCGACGTGCATTCCCGATTCCTT-3′; LDHA Reverse: 5′-GACGGCTTTCTCCCTCTTGCTGACG-3′; GPR81 Forward: 5′-AATTTGGCCGTGGCTGATTTC-3′; GPR81 Reverse: 5′-ACCGTAAGGAACACGATGCTC-3′; HIF1α Forward: 5′-TTGATGGGATATGAGCCAGA-3′; HIF1α Reverse, 5-TGTCCTGTGGTGACTTGTCC-3′; GAPDH Forward: 5′-TGTTGCCATCAATGACCCCTT-3′; and GAPDH Reverse: 5′-CTCCACGACGTACTCAGCG-3′.

Samples were quantified at the log-linear portion of the curve using LightCycler analysis software and compared with an external calibration standard curve.

### Plasmids/constructs.

For loss of function, LDHA, GPR81, and HIF1α were knocked down using shRNA (pGIPZ-LDHA, pGIPZ-GPR81, or pGIPZ-HIF1α shRNA). Scrambled shRNA served as control. Cells were transduced with LDHA, GPR81, or HIF1α shRNA or scrambled shRNA with 5 ng/mL of Polybrene (MilliporeSigma).

### Western blot and immunoprecipitation.

Western blots were performed as previously described (71). For immunoprecipitation, nuclear fractions were isolated by NE-PER Nuclear and Cytoplasmic Extraction reagents. The samples were centrifuged at 12,000*g* for 15 minutes at 4°C, and the lysates were precleared for 1 hour at 4°C with protein A/G beads and immunoprecipitated for 2 hours at 4°C with the appropriate primary antibody.

### ChIP assay.

ChIP Assay kit (Ab185913, Abcam) was used. In total, 6 ***×*** 10^6^ cells (with the treatment indicated or vehicle control) were used for chromatin fragment preparation. The experiment was conducted following the manufacturer’s instruction. ChIP-PCR was performed using GPR81 forward primer 5′-CAAGGCAGTCAGGCAAAG-3′ and GPR81 reverse primer 5′-CTTGTTTGTCCTCCCTCCT-3′.

### IHC of IPF lung tissue.

IHC was performed on 4 μm paraffin-embedded serial sectioned IPF lung tissue and mounted on polylysine-coated slides. The sections were deparaffinized in xylene, rehydrated through a graded Methanol series, quenched with 0.3% hydrogen peroxide in methanol, and immersed in a 98°C water bath for 30 minutes in citrate buffer (pH 6.0) for antigen retrieval. Sections were placed in 5% normal horse serum (Jackson ImmunoResearch) to block nonspecific binding of secondary antibodies. A multiplex IHC kit was used for antigen detection according to the manufacturer’s instructions (MULTIVIEW IHC Kit ADI-950.101.0001; Enzo Life Sciences). The tissue specimens were incubated overnight (18–20 hours, 4°C) with the following primary antibodies: anti–rabbit GPR81 monoclonal antibody (1:800) (Ab188647, Abcam), anti–human CAIX antibody (1:500) (Ab15086, Abcam), and anti–human SSEA4 antibody (1:100) (clone MC-813-70, catalog 330402, BioLegend). Specimens were cover-slipped with a Prolong Antifade Kit (Invitrogen/Molecular Probes) and stored overnight at room temperature without light before image analysis.

### In vivo assessment of fibrogenicity.

To assess IPF MPC fibrogenicity in vivo, we utilized 2 assays. Antibodies are listed in Supplemental Table 1.

### Zebrafish xenotransplant model.

Cells stained with a vital dye (5 μM/L PKH26; Sigma-Aldrich) were grafted into the central portion of the zebrafish embryo blastoderm at the oblong-sphere stages as previously described. Embryos were immobilized with Tricane after 48 hours. Fluorescence and corresponding bright-field imaging of the PKH26-stained grafts in live embryos were performed with a Zeiss Axiovert upright microscope (Carl Zeiss). After image acquisition, embryos were fixed with 4% paraformaldehyde, infiltrated with increasing concentrations of sucrose/PBS, and cryoembedded in OCT. The size of the fibrotic reticulum was quantified as previously described (41).

### Mouse xenograft model of fibrotic progression.

NSG male and female mice (The Jackson Laboratory), average 10 weeks of age, were administered low-dose intratracheal bleomycin (1.25 U/kg) (9). Two weeks following a nonfibrogenic dose of bleomycin i.t., GPR81 shRNA or scrambled shRNA IPF MPCs were suspended in PBS (1 ***×*** 10^6^ cells/100 μL) and injected via tail vein. Following cell administration, the mice were maintained under normoxic (21% O_2_) or hypoxic conditions (10%O_2_; BioSpherix hypoxia chamber) for the duration of the experiment. Mice were euthanized 4 weeks after adoptive transfer of human cells, and the lungs were harvested. Collagen content was quantified in the left lung tissue by Sircol assay and served as the primary endpoint. Histological (H&E and trichrome staining) and IHC analysis was performed on paraffin-embedded and frozen right lung tissue. The presence of lung fibrotic lesions by histological analysis served as the secondary endpoint. Cells positive for human procollagen (anti–human procollagen type I antibody, 1:500; catalog MAB1912; EMD Millipore) were identified as human. IHC using the human procollagen antibody and GPR81 antibody was performed to assess the distribution of GPR81-expressing cells with human procollagen I expressing cells. The antibody used does not cross-react with mouse collagen.

### Quantification of human IPF cells in mouse lung tissue.

Mice were euthanized 4 weeks after adoptive transfer of human cells, and the lungs were harvested. The lungs were digested, and genomic DNA was isolated using a PureLink Genomic DNA Mini Kit according to the manufacturer’s instructions (Invitrogen). Real-time PCR was used to quantify human IPF cells in the mouse lungs by measuring the amount of human-specific DNA sequence using human specific primers per a previously published protocol (44). PCR assay was performed for 40 cycles using the human genomic DNA-specific primers (forward: 5′-ATGCTGATGTCTGGGTAGGGTG-3′; reverse: 5′-TGAGTCAGGAGCCAGCGTATG-3′). Genomic DNA from 1 ***×*** 10^6^ IPF MPCs was used as reference control in qPCR.

### Statistics.

Comparisons of data among experiments were performed using the 2-tailed Student’s *t* test and 1-way ANOVA, as indicated. *P* < 0.05 was considered significant.

### Study approval.

Deidentified patient samples were obtained by our tissue procurement service (Bionet) under a waiver of informed consent from the University of Minnesota IRB (no. 1504M68341). Fresh IPF lungs were obtained at the time of lung transplantation, and control lung tissue was obtained by VATS biopsy or lobectomy. Animal protocols were approved and conducted in accordance with the University of Minnesota IACUC regulations (approval no. 1706-34890A).

## Author contributions

LY conceived, designed, and directed the studies with input from PBB and CAH. LY and CAH wrote the manuscript with assistance from all the authors. LY established primary human mesenchymal cell lines, cultured MPCs, performed flow cytometry for isolation of MPCs, performed qPCR, completed Western blot analyses, and performed gain- and loss-of-function experiments, mouse studies, and IHC. AG and HX assisted with isolation and culture of MPCs and assisted with mouse studies. ER provided slides for IHC. HX and JH also assisted with IHC analyses. KS designed and constructed expression constructs, and AB performed zebrafish xenotransplantation of MPCs.

## Supplementary Material

Supplemental data

## Version 1 01/19/2023

In-Press Preview

## Version 2 02/22/2023

Electronic publication
