# Supplementary material for: Hypoxia enhances IPF mesenchymal progenitor cell fibrogenicity via the lactate/GPR81/HIF1α pathway
Source: JCI Insight. 2023 Feb 22;8(4):e163820. doi: 10.1172/jci.insight.163820 (PMC9977506; doi:10.1172/jci.insight.163820)
Supplement: Supplemental data [file jciinsight-8-163820-s111.pdf]

SUPPLEMENTARY FIGURES

Supplementary Figure 1

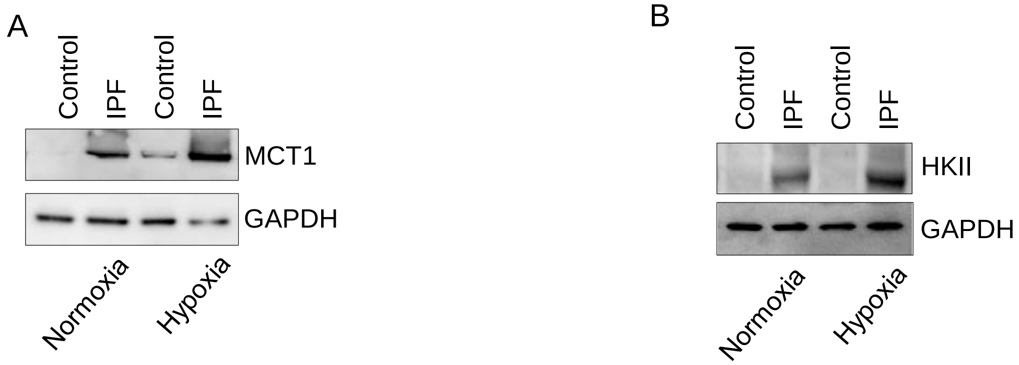

Supplementary Figure 1. Western blot analysis of glycolysis marker MCT1 and HKII. **A and B.** IPF MPCs were cultured under hypoxia (2% O2) or normoxia conditions for 24 hours, MCT1 (**A**) and HKII (**B**) expression were quantified by Western blot analysis. GAPDH served as a loading control.

Supplementary Figure 2

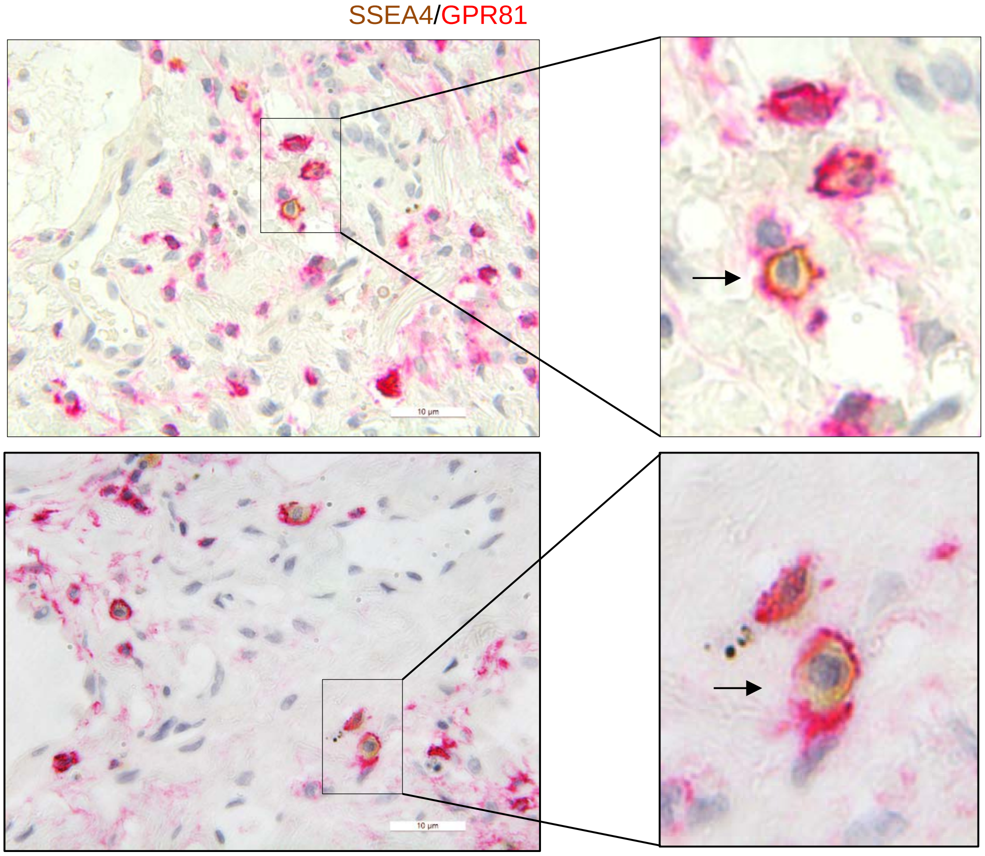

Supplementary Figure 2. SSEA4+ IPF MPCs express the lactate receptor GPR81. IHC double staining was performed on IPF lung tissue using GPR81 (red) and SSEA4 (brown-yellow) antibodies. Shown are IPF MPCs staining positive for both SSEA4 and GPR81 from two different areas. Scale bar = 10 µm.

Supplementary Figure 3

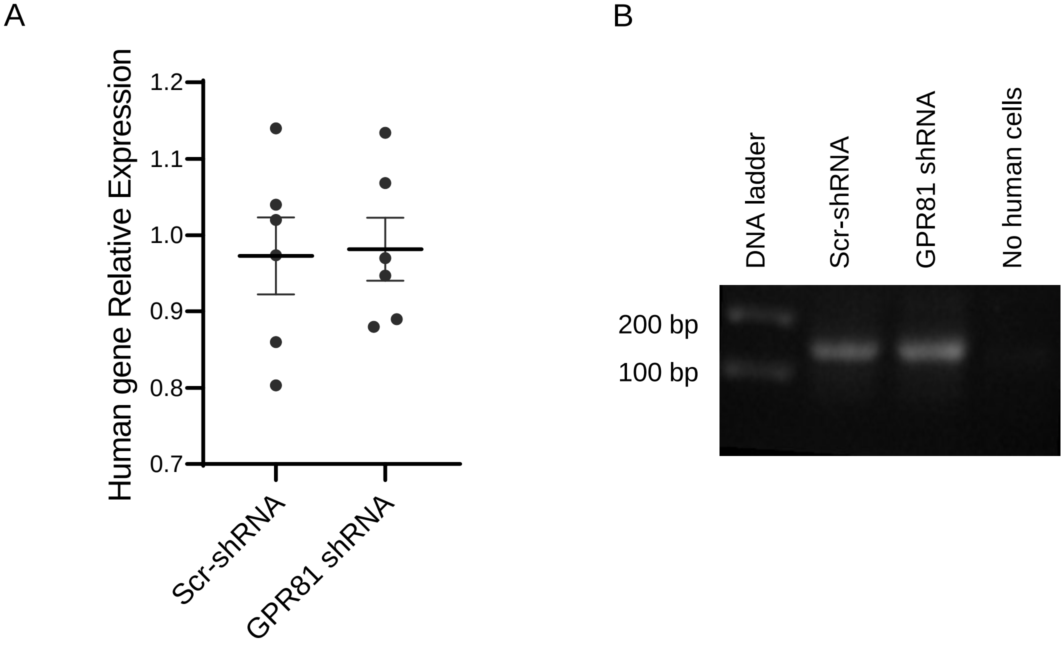

Supplementary Figure 3. Confirmation of engraftment of human scrambled shRNA and GPR81 shRNA IPF MPCs in immune-deficient mice by real-time PCR. Three groups of immune-deficient mice (n=2 each) were administered intratracheal bleomycin (1.25 U/kg). In 2 groups of mice, 10^6 IPF MPCs were injected via tail vein 2 weeks after intratracheal bleomycin. The third group did not receive human cells and served as a negative control. Seven days after administration of cells the lungs were harvested for analysis of engraftment of human cells. The lungs were digested and genomic DNA isolated using a PureLink Genomic DNA Mini Kit according to the manufacturer's instructions (Invitrogen, Carlsbad,CA). Real-time PCR was used to quantify human IPF cells in the mouse lungs by measuring the amount of human-specific DNA sequence using human specific primers per a previously published protocol (38). PCR assay was performed for 40 cycles using the human genomic DNA-specific primers (forward: ATGCTGATGTCTGGGTAGGGTG; reverse: TGAGTCAGGAGCCAGCGTATG). A. Engraftment of human IPF MPCs was quantified by Q-PCR. B. Shown is gel electrophoresis of the PCR products. A single, predicted 141 base pair band specific for the human genomic DNA sequence was amplified from the DNA extracted from the lungs of mice receiving human cells (lanes 3 and 4). Lane 1: DNA ladder. Lane 2: mouse lung containing human scrambled shRNA IPF MPCs. Lane 3: mouse lung containing human GPR81 shRNA IPF MPCs. Lane 4: mouse lung only (no human cells).

Supplementary Figure 4

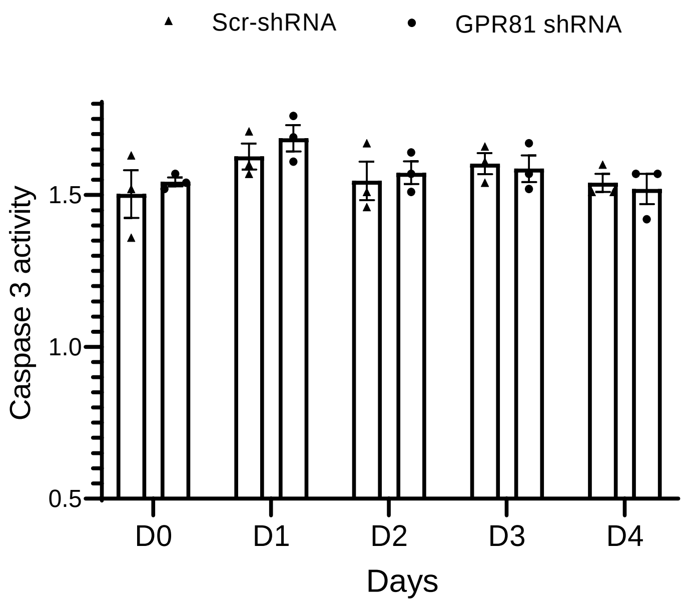

Supplementary Figure 4. IPF MPCs were transduced with GPR81 shRNA or scrambled shRNA and cultured under hypoxic conditions (10% O2). Caspase 3 levels were quantified at the indicated times.

Supplementary Figure 5

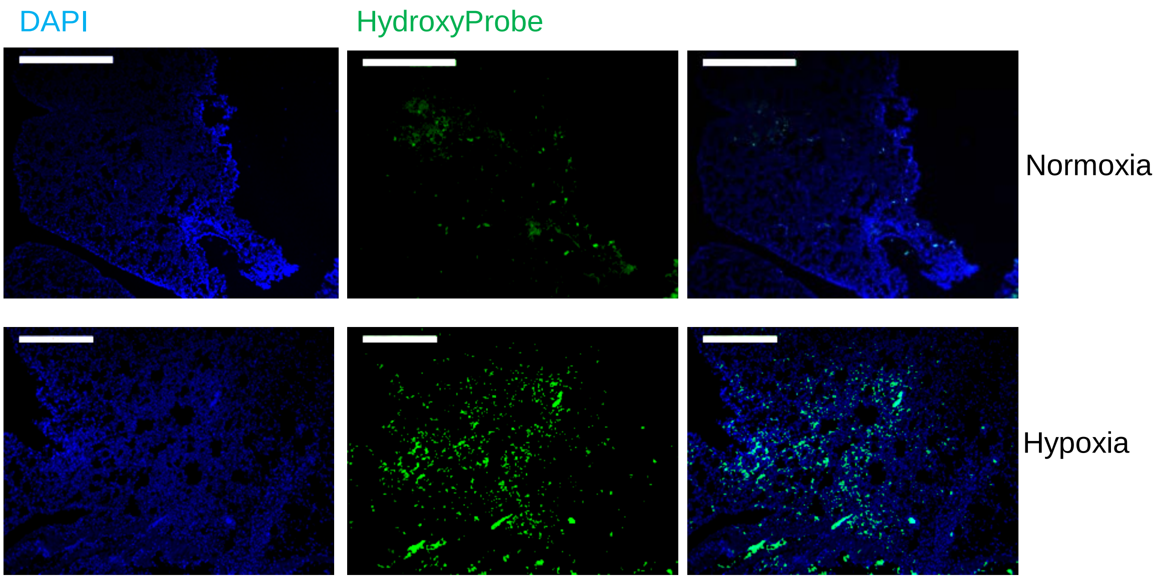

Supplementary Figure 5. Immunohistofluorescence of mouse lung tissue using the hydroxyprobe reagent kit to identify hypoxic tissue. NSG mice were administered bleomycin IT followed 2 weeks later by IPF MPCs. The mice were maintained in normoxic or hypoxic conditions for the duration of the experiment (4 weeks). Just prior to euthanasia, the hydroxyprobe reagent (pimonidazole hydrochloride) was administered IP. Immunohistofluorescence of harvested lung tissue was performed according to the manufacturer's instructions. Left hand panels: DAPI stain demonstrating cell nuclei (blue). Middle panels: Hydroxyprobe stain demonstrating hypoxic tissue (green). Right hand panels: DAPI plus hydroxyprobe staining. Top row: normoxia (21% O2); Bottom row: hypoxia (10% O2). Scale bars: 500 µm.

Supplementary Figure 6

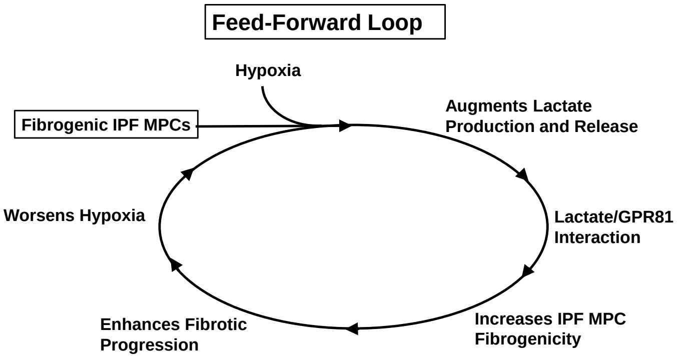

Supplementary Figure 6. **Hypoxia mediated feed-forward loop in IPF MPCs**. IPF MPCs are intrinsically fibrogenic. Under normoxic conditions they display increased lactate levels compared to control MPCs. Hypoxia, which is a prominent clinical feature of IPF pathophysiology augments IPF MPC lactate production and release and increases HIF1α expression. HIF1α enhances expression of the lactate receptor GPR81. Increased lactate, operating through the GPR81, augments IPF MPC fibrogenicity and enhances fibrotic progression. Thus, hypoxia creates a feed-forward loop that enhances lactate production in IPF MPCs and augments their fibrogenicity.
